# Balloon pulmonary valvuloplasty for severe rheumatic pulmonary stenosis in quadrivalvular heart disease after prior multivalve surgery: a case report

**DOI:** 10.1093/ehjcr/ytag334

**Published:** 2026-05-09

**Authors:** Tsebaot Tesfaye, Elsa Wolde Mamo, Mohammed Bedru

**Affiliations:** Department of Cardiovascular Medicine, Addis Ababa University, P.O.Box 9086, Addis Ababa 1000, Ethiopia; Addis Ababa University, College of Health Sciences, School of Medicine, P.O.Box 9086, Addis Ababa 1000, Ethiopia; Cardiac Center—Ethiopia, Addis Ababa 1000, Ethiopia

**Keywords:** Rheumatic heart disease, pulmonary stenosis, quadrivalvular involvement, balloon pulmonary valvuloplasty, secondary prophylaxis, echocardiography, case report

## Abstract

**Background:**

Rheumatic heart disease predominantly affects left-sided cardiac valves. Quadrivalvular involvement with clinically significant pulmonary valve stenosis is exceedingly rare, and evidence regarding minimally invasive management strategies in such complex cases is limited.

**Case summary:**

A 30 year-old woman with chronic rheumatic heart disease and prior mitral and aortic bioprosthetic valve replacement with tricuspid valve repair presented with a four-month history of progressive exertional dyspnea and palpitations (New York Heart Association functional class lll). Transthoracic echocardiography revealed severe pulmonary valve stenosis with a peak gradient of 97 mmHg, moderate pulmonary regurgitation, mild tricuspid regurgitation, right ventricular hypertrophy with preserved systolic function, and normally functioning prosthetic mitral and aortic valves. Given the presence of predominant commissural fusion, minimal valve calcification, preserved right ventricular function, and elevated surgical risk due to prior cardiac surgery, percutaneous balloon pulmonary valvuloplasty was performed. An 18 x 40 x 110 mm Nucleus balloon was deployed under fluoroscopic guidance, resulting in immediate symptomatic improvement. Post-procedural echocardiography demonstrated a reduction in the pulmonary valve gradient to 35 mmHg. At follow-up visit at 1 week, 1 month, and 3 months, the patient remained asymptomatic, with sustained gradient reduction (35-38 mmHg), preserved right ventricular function, moderate but clinically well-tolerated pulmonary regurgitation, and continued normal prosthetic valve function.

**Discussion:**

Pulmonary valve involvement in rheumatic heart disease is rare and typically occurs as part of multivalvular disease. In carefully selected patients, balloon pulmonary valvuloplasty can provide a safe and effective minimally invasive alternative to reoperation, achieving durable hemodynamic and clinical improvement.

Learning points

- Balloon pulmonary valvuloplasty can be an effective minimally invasive treatment for severe rheumatic pulmonary stenosis in carefully selected adult patients.
- Comprehensive monitoring of all four heart valves is essential in cases of rheumatic heart disease, even following surgery on the left-sided valves.
- Strict adherence to secondary penicillin prophylaxis is crucial for preventing recurrent acute rheumatic fever and limiting the progression or new involvement of rheumatic valvular disease.

## Introduction

Rheumatic heart disease (RHD) continues to be a leading cause of acquired valvular disease worldwide, particularly in low- and middle-income countries.^1^ It primarily affects the mitral and aortic valves, while right-sided valvular involvement is rare. Quadrivalvular disease, which may include pulmonary valve stenosis, is exceptionally uncommon. In cases of pulmonary valve disease associated with RHD, it is typically managed surgically.

Balloon pulmonary valvuloplasty (BPV) is an established treatment for congenital pulmonary stenosis and is recommended by guidelines for adults with appropriate valve morphology.^2^ However, reports of BPV being used for acquired or rheumatic pulmonary stenosis are limited.^3,4^

In this report, we present a rare case of severe pulmonary stenosis in a patient with quadrivalvular RHD, successfully managed with BPV despite the patient’s history of previous multivalvular surgery. This case offers valuable insights for cardiologists treating advanced RHD with right-sided involvement in adults.

## Summary figure

**Figure:**
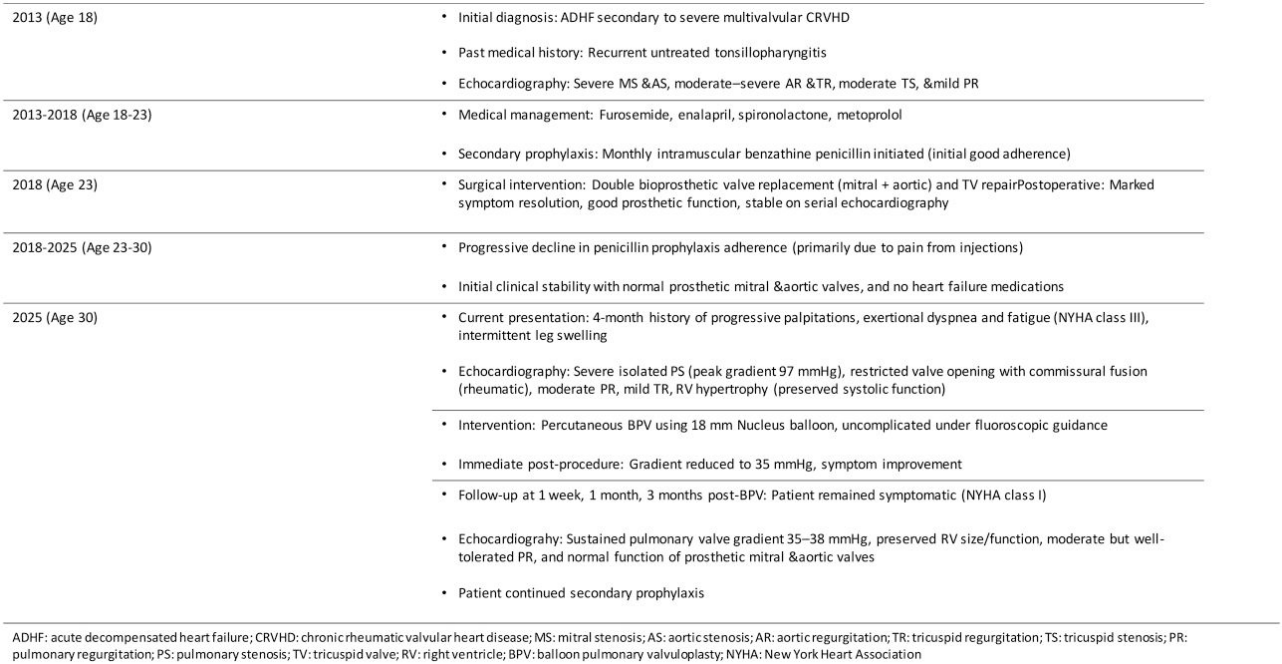
Timeline illustrating key clinical events in a 30-year-old woman with chronic rheumatic heart disease. The timeline shows initial diagnosis at age 18 with severe multivalvular involvement, subsequent valve surgery, and a period of declined adherence to secondary benzathine penicillin prophylaxis. By age 30, the patient developed severe pulmonary valve stenosis, which was successfully treated with percutaneous balloon pulmonary valvuloplasty, resulting in sustained haemodynamic and symptomatic improvement at 3-month follow-up.

## Case presentation

A 30-year-old woman with a known history of chronic rheumatic heart disease (CRHD) involving multiple valves presented with a 4-month history of progressive palpitations, exertional dyspnoea, and fatigue [New York Heart Association (NYHA) functional class III]. She also reported intermittent lower-extremity oedema but denied chest pain, syncope, orthopnoea, or paroxysmal nocturnal dyspnoea.

She was first diagnosed with CRHD at 18 years of age following an episode of decompensated heart failure. Her past medical history included recurrent episodes of inadequately treated tonsillopharyngitis and limited access to healthcare. She did not recall any documented episodes of acute rheumatic fever and had no significant comorbidities or family history of cardiac disease. Initial transthoracic echocardiography demonstrated severe mitral stenosis, severe aortic stenosis with moderate-to-severe aortic regurgitation, moderate tricuspid stenosis with moderate-to-severe tricuspid regurgitation, mild pulmonary regurgitation, and mild pulmonary hypertension. She experienced multiple heart failure admissions over subsequent years and was managed medically with furosemide, enalapril, spironolactone, and metoprolol. Monthly intramuscular benzathine penicillin was initiated for secondary prophylaxis, with good initial adherence.

Seven years prior to the current presentation (5 years after diagnosis), she underwent double bioprosthetic valve replacement of the mitral and aortic valves with concomitant tricuspid valve repair. Postoperative echocardiography showed well-functioning prosthetic valves with significantly reduced gradients, and she experienced marked clinical improvement with resolution of heart failure symptoms. Serial follow-up echocardiograms confirmed stable prosthetic valve function and no significant progression of residual valvular disease.

Following the surgery, her adherence to benzathine penicillin gradually declined due to injection-related pain, and her heart failure medications were tapered as she remained clinically stable on low-dose aspirin. Although she did not experience any documented episodes of acute rheumatic fever, intermittent tonsillopharyngitis persisted.

**Figure 1:**
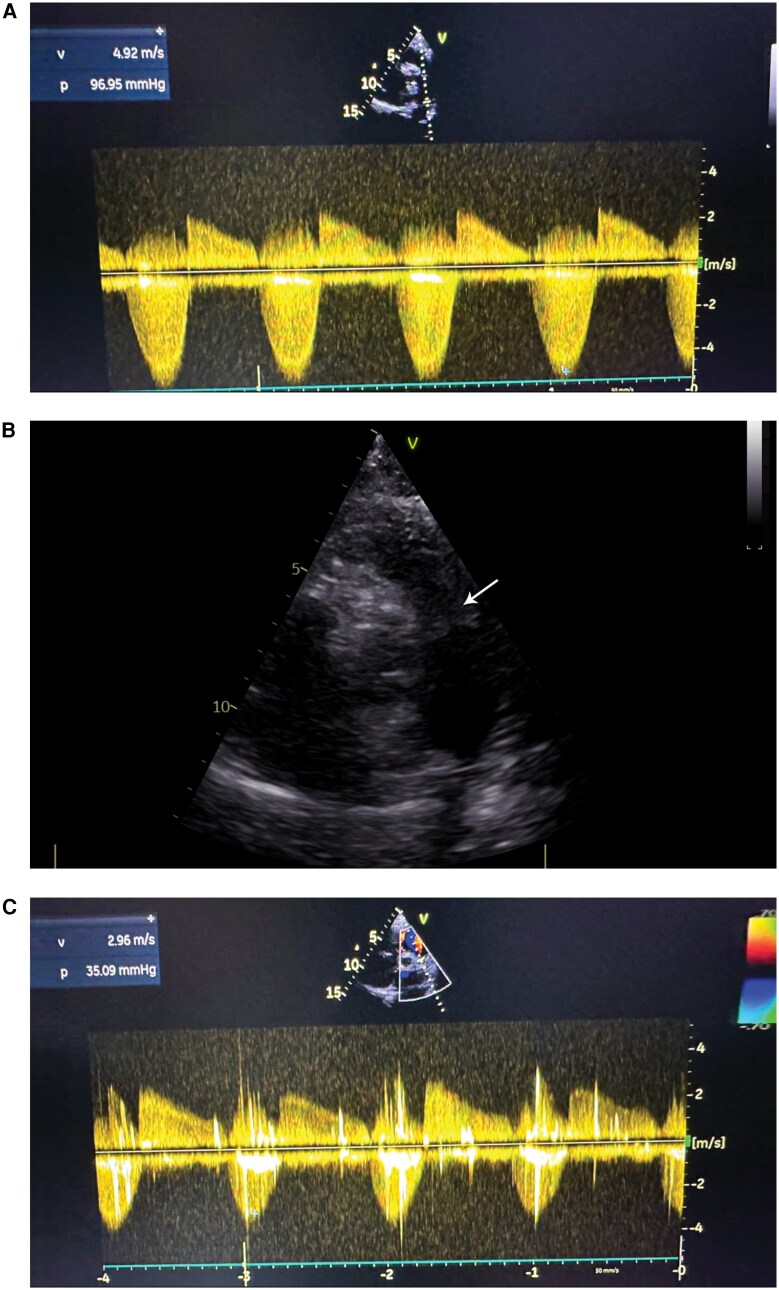
Transthoracic echocardiography of the pulmonary valve before and after balloon pulmonary valvuloplasty. (*A*) Continuous-wave Doppler echocardiography across the pulmonary valve demonstrating severe pulmonary stenosis with a peak gradient of 97 mmHg prior to intervention. (*B*) Parasternal short-axis echocardiographic view of the pulmonary valve demonstrating restricted systolic opening with commissural fusion (arrow). See Supplementary material online, *Video*  S1 for dynamic visualization. (*C*) Continuous-wave Doppler echocardiography across the pulmonary valve following balloon pulmonary valvuloplasty showing a marked reduction in the peak gradient to 35 mmHg.

On presentation, her heart rate was 65 beats/min and blood pressure 110/70 mmHg. Jugular venous pressure was normal, and there was no peripheral oedema. Cardiac auscultation revealed a harsh systolic ejection murmur with an ejection click best heard at the left upper sternal border. Transthoracic echocardiography demonstrated severe pulmonary valve stenosis with a peak gradient of 97 mmHg, moderate pulmonary regurgitation, and mild tricuspid regurgitation (*Figure 1A*). Parasternal short-axis imaging showed restricted pulmonary valve opening with commissural fusion, consistent with rheumatic involvement (*Figure 1B*; Supplementary material online, *Video*  S1). The right ventricle was hypertrophied with preserved systolic function, and both prosthetic mitral and aortic valves functioned normally.

**Figure 2:**
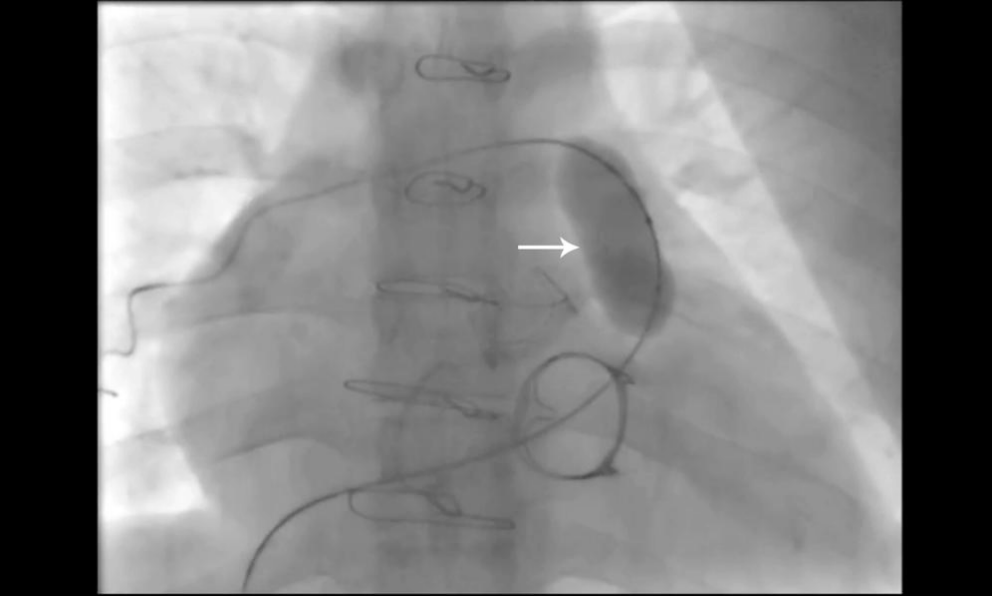
Fluoroscopic image (RAO projection) during balloon pulmonary valvuloplasty. An 18 × 40 × 110 mm nucleus balloon positioned and fully inflated across the pulmonary valve with complete disappearance of the waist (arrow). Previously replaced mitral and aortic valves and repaired tricuspid valve are visible. Multiple sternal wires from prior surgery are also seen along the sternum. See Supplementary material online, *Video*  S2 for full procedure.

Given severe symptomatic pulmonary stenosis, prior cardiac surgeries, predominant commissural fusion with minimal calcification, preserved right ventricular function, and low procedural risk, percutaneous BPV was performed. The pulmonary annulus measured 18 mm on transthoracic echocardiography (no pre-procedural CT was performed due to adequate windows). An 18 × 40 × 110 mm nucleus balloon was chosen for a balloon-to-annulus ratio of ∼1.0–1.1 with stepwise inflation. Potential post-dilatation pulmonary regurgitation was anticipated, and bailout strategies included close haemodynamic monitoring with initial conservative management, reserving transcatheter pulmonary valve implantation as a future option if significant regurgitation occurred. The balloon was positioned across the valve under fluoroscopic guidance and dilated to maximal size [*Figure 2*, Supplementary material online, *Video*  S2].

The procedure was uneventful, and the patient reported immediate symptomatic improvement. Post-procedure echocardiography confirmed reduction of the pulmonary gradient to 35 mmHg and preserved right ventricular function [*Figure 1B*]. She was discharged the following day in stable condition.

On follow-up at 1 week, 1 month, and 3 months, the patient remained asymptomatic (NYHA I), with sustained reduction of the pulmonary gradient (35–38 mmHg), preserved right ventricular function without dilation or dysfunction, moderate but clinically tolerated pulmonary regurgitation, and no prosthetic valve dysfunction. Planned follow-up includes clinical review and echocardiography at 6 months, 12 months, and annually thereafter, focusing on pulmonary gradient, regurgitation, RV function, and prosthetic valves. Lifelong secondary penicillin prophylaxis will be reinforced, with diuretics as needed.

## Discussion

RHD results from an abnormal immune response to infection with group A Streptococcus, leading to valvular deformities, commissural fusion, and progressive dysfunction.^3^ While the left-sided heart valves are most commonly affected, right-sided valve involvement is rare, and quadrivalvular disease is exceptionally uncommon.^5^ Pulmonary valve stenosis secondary to RHD has been reported only infrequently.^4,6^

Management of pulmonary valve disease due to RHD traditionally involves surgical repair or replacement.^7,8^ In contrast, BPV is a well-established treatment for congenital pulmonary stenosis, with proven immediate and long-term efficacy.^9–11^ Reports of BPV in acquired or rheumatic pulmonary stenosis remain limited, and disease-specific outcome data are scarce.^4,6^

Several factors influence the suitability of BPV in RHD-related pulmonary stenosis, including valve morphology, the extent of commissural fusion vs. calcification, severity of pulmonary regurgitation, and the presence of multivalvular disease.^4,12^ In patients with predominant commissural fusion and minimal calcification or regurgitation, BPV can provide effective symptomatic relief and haemodynamic improvement, potentially avoiding repeat open-heart surgery in high-risk re-operative patients. This approach is consistent with guideline recommendations for balloon valvuloplasty in severe pulmonary stenosis with suitable morphology.^2^ Surgical intervention remains preferred for severe calcification or significant regurgitation. Right heart catheterization was not performed, as echocardiography showed only mild pulmonary hypertension with dominant valvular stenosis and preserved RV function. In similar cases, it could help differentiate pre- from post-capillary pulmonary hypertension and guide potential pharmacological therapy.

Despite pre-existing moderate pulmonary regurgitation in this case, BPV was pursued as the stenotic component predominantly drove symptoms and RV hypertrophy, with preserved ventricular function and favourable valve anatomy. This decision aligns with current guidelines, which prioritize relief of severe obstruction (peak gradient >64 mmHg) in symptomatic patients, without listing mild-to-moderate pre-existing PR as a contraindication.^2^ Moderate residual pulmonary regurgitation was well-tolerated short-term in this case, with no right ventricular dilation or dysfunction at 3 months; however, longer-term surveillance is warranted due to potential progression.

This case underscores the importance of comprehensive surveillance of all four cardiac valves, as pulmonary valve disease may progress despite prior interventions on other valves. Secondary penicillin prophylaxis remains essential to prevent recurrent acute rheumatic fever and limit overall progression of rheumatic valvular disease. Regular echocardiographic follow-up is also critical to detect early right ventricular remodelling or dysfunction, which carry significant prognostic implications in multivalvular disease.

## Conclusion

Balloon pulmonary valvuloplasty can be a feasible and effective minimally invasive option for severe pulmonary stenosis secondary to RHD in carefully selected patients, particularly when commissural fusion predominates and calcification is limited. This approach offers a valuable alternative to repeat surgery in complex reoperative cases and highlights the importance of vigilant multivalvular surveillance and sustained secondary prophylaxis to optimize long-term outcomes.

## Supplementary Material

ytag334_Supplementary_Data

## Data Availability

The data supporting this case report are available from the corresponding author upon reasonable request. Due to ethical and patient privacy considerations, raw clinical data (including imaging files and identifiable information) cannot be made publicly available. Relevant de-identified clinical data, including echocardiographic measurements and procedural details, are provided within the article and its Supplementary material.

## Lead author biography

Dr. Tsebaot Tesfaye is an internist and adult cardiology fellow at Addis Ababa University, Ethiopia. She earned her MD from Addis Ababa University and completed her Internal Medicine residency at the University of Gondar, where she later served as an assistant professor, consultant internist, and postgraduate coordinator. Her interests include cardiovascular and cardio-metabolic disorders, and the use of point-of-care ultrasound (POCUS) in training and patient care. She has co-authored several international publications and engages in collaborative research. Dr. Tesfaye is a member of the Ethiopian Society of Internal Medicine, Ethiopian Society of Cardiac Professionals, and the European Society of Cardiology.

## Notes

**Consent:** Written informed consent was obtained from the patient for the publication of this case report and any accompanying images. A copy of the written consent is available for review by the Editor-in-Chief of this journal upon request.
