# Supplementary material for: Balloon pulmonary valvuloplasty for severe rheumatic pulmonary stenosis in quadrivalvular heart disease after prior multivalve surgery: a case report
Source: Eur Heart J Case Rep. 2026 May 9;10(5):ytag334. doi: 10.1093/ehjcr/ytag334 (PMC13215467; doi:10.1093/ehjcr/ytag334)
Supplement: ytag334_Supplementary_Data [file ytag334_supplementary_data.zip › Video legends.docx]

**Video Legends**

Video 1. Parasternal short-axis echocardiographic view of the pulmonary valve before intervention: Real-time transthoracic echocardiographic parasternal short-axis view demonstrating restricted pulmonary valve leaflet motion with commissural fusion.

Video 2. Fluoroscopic balloon pulmonary valvuloplasty: Fluoroscopic video demonstrating an 18 × 40 × 110 mm Nucleus balloon inflation across the pulmonary valve during percutaneous balloon pulmonary valvuloplasty. Previously replaced mitral and aortic valves and repaired tricuspid valve, and multiple sternal wires from prior surgery are visible.
